# Personal

**Published:** 1905-05

**Authors:** 


### PERSONAL.

---

Dr. E. C. Dudley, of Chicago, president of the American Gynecological Society, recently visited Buffalo, to attend a meeting of the council of that society, the object of which was in part to perfect plans for the annual meeting of the society at Niagara Falls.

---

Dr. Charles F. Durand, of Buffalo, has been appointed superintendent of the quarantine hospital vice A. T. O'Hara, deceased. Dr. Durand stood highest on the civil service list, from which the appointment was made and which carries a salary of $1,200 a year.

---

Dr. and Mrs. Dewitt C. Greene, of Buffalo, sailed for Europe from Boston, on the White Star steamer Romanic, April 22, 1905. They will be absent about three months.

---

Dr. A. A. Hubbell, of Buffalo, recently was admitted to Fellowship in the New York Academy of Medicine.

---

Dr. J. Henry Dowd, of Buffalo, has removed from 378 Franklin street to The Raleigh, 354 Franklin street. Hours: 9 to 1. Sundays, 2 to 4. Both telephones.

---

Dr. Lewellys Franklin Barker, of Chicago, has been elected by the trustees of Johns Hopkins University to be professor of medicine in that institution.

---

Dr. Henry C. Buswell, of Buffalo, has removed from 868 Main street, to 1342 Main street, near Utica.

---

Hon. Henry W. Hill, State Senator from the forty-seventh district (Buffalo) is making a three months' tour of Europe. He is accompanied by Mrs. Hill and expects to return about July 25.

---

Dr. William H. Marcy, of Buffalo, has been appointed surgeon to the fire department vice Dr. E. C. W. O'Brien, deceased. This selection was made from the civil service list to take effect May 1. The salary is $1,500 a year.

